# Comparison of the GEM and the ECAL indirect calorimeters against the Deltatrac for measures of RMR and diet-induced thermogenesis

**DOI:** 10.1017/jns.2014.58

**Published:** 2014-11-07

**Authors:** S. Kennedy, L. Ryan, A. Fraser, M. E. Clegg

**Affiliations:** 1 Department of Sport and Health Sciences, Faculty of Health and Life Sciences, Oxford Brookes University, Functional Food Centre, Gipsy Lane, Oxford OX3 0BP, UK; 2 Department of Nutrition and Dietetics, Faculty of Medicine, Nursing and Health Sciences, Monash University, 264 Ferntree Gully Road, VIC 3168, Australia

**Keywords:** RMR, Diet-induced thermogenesis, GEM, ECAL, Deltatrac, CHO, carbohydrate, DIT, diet-induced thermogenesis, EE, energy expenditure, GEM, gas exchange measurement, MCT, medium-chain TAG, RQ, respiratory quotient, T1, test day 1, T2, test day 2

## Abstract

The Deltatrac™ II Metabolic Monitor (Datex-Ohmeda Inc.) is considered the standard reference machine in indirect calorimetry; however, it is no longer commercially available thus there is a need for new machines. The gas exchange measurement (GEM; GEM Nutrition Ltd) and the ECAL (Health Professional Solutions) are alternative measuring systems. The aim of this study was to compare the ECAL and GEM with Deltatrac for measures of RMR and the GEM to the Deltatrac for measures of diet-induced thermogenesis (DIT). Twenty healthy participants were tested on test day 1 (T1) and test day 2 (T2). RMR was measured in a randomised order for 30 min on the Deltatrac, the GEM and the ECAL. Following this, a 1553 kJ meal was consumed and DIT was measured on the Deltatrac and the GEM in alternating 15 min intervals for 4 h. The GEM reported consistently higher values than the Deltatrac for V_O2_, V_CO2_, RMR and fat oxidation (*P* < 0·005). The ECAL was significantly higher than the Deltatrac for measures of VO_2_, RMR, carbohydrate oxidation (T2) and respiratory quotient and fat oxidation (T1, T2) (*P* < 0·05). There were no significant differences within repeated RMR measures on the ECAL, the GEM or the Deltatrac. DIT measures were consistently higher on the GEM (T1) (*P* < 0·005); however, there were no significant differences between repeated measures. The findings suggest that while the GEM and the ECAL were not accurate alternatives to the Deltatrac, they may be reliable for repeated measures.

Indirect calorimetry is a method used to estimate RMR and diet-induced thermogenesis (DIT) from measurements of V_O2_ and carbon dioxide production (V_CO2_)^(^^1^^)^. RMR can account for 45–70 % of daily total energy expenditure (EE) and DIT about 10 %, based on consuming a variety of foods^(^^2^^)^. Measures of EE are fundamental to nutritional assessments in clinical and research settings. The use of indirect calorimetry provides more accurate measures compared with predictive equations which have been reported to over or under estimate in some populations^(^^3^^–^^7^^)^. However, indirect calorimeters are expensive therefore these equations remain in use in a number of clinical settings.

In a clinical setting the precision of measurements on indirect calorimeters is critical as these data are primarily used for determining the energy requirements of patients to prevent under or over feeding^(^^7^^)^. In a research or weight-loss setting users often measure differences in metabolic rate over time^(^^8^^)^ or following an intervention^(^^9^^)^. In these circumstances, the accuracy of repeated measures on the same machine (i.e. repeatability) is of upmost importance.

For over 24 years the Deltatrac™ II Metabolic Monitor (Datex-Ohmeda Inc.) has been considered the standard reference tool in indirect calorimetry. It has been validated *in vitro* and *in vivo* for use in clinical and research settings^(^^10^^,^^11^^)^ and is therefore often used as a comparison in many validation studies^(^^12^^–^^21^^)^. Now that the Deltatrac is no longer commercially available there is increasing interest in alternative indirect calorimeters^(^^12^^–^^21^^)^. An attractive alternative to the Deltatrac would be less expensive and require minimal technical expertise for maintenance and use. Furthermore, mobile indirect calorimeters are appealing to wider audiences who require greater accuracy in the field and a machine that can be easily transported. Numerous studies have compared indirect calorimeters against the Deltatrac using both portable and hooded versions for measures of RMR^(^^12^^,^^14^^–^^18^^,^^20^^–^^24^^)^, and to a lesser extent, DIT^(^^13^^,^^19^^)^. Conclusions have been conflicting with some studies reporting agreement between methods^(^^12^^,^^13^^,^^15^^,^^18^^,^^19^^,^^21^^)^ and other studies rejecting agreement^(^^14^^,^^16^^,^^17^^,^^22^^)^.

Making comparisons between machines can be problematic due to differences in how measures are collected, in particular, between the portable calorimeters and the hooded canopies. Indirect calorimeters that use a ventilated hood such as the Deltatrac are often set to generate data from V_O2_ and V_CO2_ samples at 1 min intervals. In contrast, other calorimeters, such as the Cosmed K4 b^2^ are able to perform a measurement on every breath^(^^16^^)^. Measures collected also vary between machines, with some calorimeters such as the MedGem RMR^®^ (Microlife), collecting V_O2_ and subsequently calculating V_CO2_ using an assumed value of 0·85 for the respiratory quotient (RQ). Consistently, most machines use a modified Weir equation to calculate RMR^(^^21^^)^.

A potential replacement for the Deltatrac is the gas exchange measurement (GEM, GEM Nutrition Ltd). Similar to the Deltatrac, the GEM is an open circuit indirect calorimeter using a ventilated hood. A clear plastic hemispherical canopy is placed over the participant's head and the exhaled air is drawn through a Nafion tube into a mixing chamber. Here, it is diluted with the room air before V_O2_ and V_CO2_ sensors collect a sample. In contrast to the Deltatrac, which assumes a constant flow of air, the GEM measures the actual flow rate through the hood, which can be varied in the range of 20 to 80 litres/min. Like the Deltatrac, the GEM measures V_O2_ using a paramagnetic oxygen sensor and V_CO2_ using an IR sensor. Data on the GEM are also reported at 1 min intervals.

A potential portative replacement to the Deltatrac is the ECAL (Health Professional Solutions). The ECAL is a novel open circuit portable indirect calorimeter that measures both V_O2_ and V_CO2_ using a small mixing chamber. The ECAL was primarily designed to be used by health professionals and therefore is low maintenance and requires little technical expertise. The machine uses a proprietary mouth piece (patent pending) and nose clip. V_O2_ is measured using a galvanic fuel cell oxygen analyser. V_CO2_ is measured using a patented ultra-low power V_CO2_ analyser which uses light emitting diode and detector technology in a novel non-dispersive near IR absorption sensor. Data on the ECAL are reported for each breath cycle.

The present study had two main outcomes. The first was to compare two new indirect calorimeters (the ECAL and GEM) with the Deltatrac for measures of RMR. The second was to compare the GEM with the Deltatrac for measures of DIT. To our knowledge this was the first study to compare RMR measures from the ECAL and the GEM to the Deltatrac, and, the first study to compare the GEM to the Deltatrac for measures of DIT.

## Methods

The present study was a randomised, crossover study with repeated measures on two different days within a 2-week time period.

### Subjects

Twenty healthy participants (four males and sixteen females) were recruited for the study (Table 1) by means of advertisements displayed throughout Oxford Brookes University and classified advertisements on a local website. Before inclusion potential participants were briefed on all aspects of the study and were given the opportunity to ask questions. Individuals fulfilling all eligibility criteria (age 18–55 years, BMI between 18 and 30 kg/m^2^, blood pressure 110–120/75–85 mmHg, not on prescription medication, no known genetic or metabolic diseases, no food allergies or intolerances) were included in the study.

**Table 1.** Participant characteristics collected on test day 1 and test day 2 (Mean values and standard deviations)

	Males (*n* 4)		Females (*n* 16)		All (*n* 20)	
	Mean	sd	Mean	sd	Mean	sd
Age (years)	38·3	11·2	24·1	3·5	26·9	8·0
Height (m)	1·8	0·04	1·7	0·1	1·69	0·1
Weight (kg)	81·8	7·1	62·3	9·4	66·2	11·9
BMI (kg/m^2^)	24·4	1·4	22·6	2·6	22·9	2·5
Fat mass (kg)	12·7	5·9	18·1	5·9	17	6·1
Fat-free mass (kg)	69·1	7·5	44·2	4·0	49·2	11·2
Body fat (%)	15·4	6·6	28·4	5·3	25·8	7·6

The study was conducted at the Functional Food Centre at Oxford Brookes University. On the day prior to each test, participants were asked to abstain from alcohol and caffeine, and to refrain from strenuous physical activity. Participants arrived between 07·00 and 09·00 hours after an overnight fast (10–12 h before testing time). A record was made of their evening meal and participants were asked to repeat this the evening prior to their next testing session. The present study was conducted according to the guidelines laid down in the Declaration of Helsinki and all procedures involving human subjects were approved by the University Research Ethics Committee at Oxford Brookes University. Written informed consent was obtained from all participants.

### Study design

**Fig. 1.**
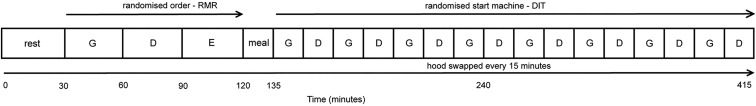
On arrival at the laboratory (time 0) participants rested for 30 min (rest). In a randomised order participant's RMR was consecutively measured for 30 min on the GEM (G), the Deltatrac (D) and the ECAL (E). Following this, participants had 15 min to consume a 1553 kJ standardised meal (meal). Diet-induced thermogenesis (DIT) was measured in a randomised start order on the GEM (G) and the Deltatrac (D) alternating the hoods in 15 min intervals for the remaining 4 h until the end of the study (415 min).

Each testing day was split into two testing phases, (1) RMR and (2) DIT (Fig. 1). The RMR phase collected measures of V_O2_ and V_CO2_ from three indirect calorimeters (the Deltatrac, GEM and ECAL). The DIT phase collected the same measures from two indirect calorimeters (the Deltatrac and GEM) following the ingestion of a 1553 kJ meal.

### Deltatrac

The Deltatrac was calibrated once in the morning after it had warmed up for 30 min. Gas was calibrated using standard calibration gases (Quick Cal) to 5 % CO_2_ and 95 % O_2_. Pressure was measured then adjusted to match ambient air pressure as reported in the daily weather forecast. Measurements were collected using a ventilated hood and artefact suppression was switched off. The flow rate was fixed at 40 litres/min; therefore no manual adjustment was required.

### Gas exchange measurement

The GEM was calibrated after a 20 min warm up. Calibrations were performed before each use, when the machine was left idle for more than 20 min, and after every 2 h of continuous use (as per the manufacturer's recommendation). Gas was calibrated using standard calibration gases (Boc) to 1 % CO_2_ and 20 % O_2_. Flow was manually adjusted at the time of calibration to approximately 40 litres/min and measurements were collected using a ventilated hood. Monthly ethanol burning tests were performed on the GEM as a quality check for the calibration.

### ECAL

The ECAL was calibrated once in the morning after a 10 min warm up. Gas was calibrated using standard calibration gas (Calgaz) to 4 % CO_2_ and 16 % O_2._ Flow was calibrated using a 1 litre calibration syringe (Hans Rudolph Series 5540). ECAL uses a proprietary method for calculating individualised flow rates with a set upper limit of 4 litres/min.

With the exception of the ECAL all calibrations were performed in line with the manufacturer's recommendations. The latest ECAL User Manual^(^^25^^)^ recommends regular calibration of the sensors (i.e. weekly) are performed; however, calibrations were performed on every test day to maintain consistency between protocols. Calibration values were checked either directly with the manufacturers (GEM and ECAL) or against manufacturer's guidelines (Deltatrac) and were found to be within acceptable ranges.

### Study protocol

On arrival at the laboratory anthropometric measures were taken. Body weight and percentage body fat were collected using the Tanita BC-418 MA (Tanita UK Ltd). Height was measured to the nearest centimetre using a stadiometer (Seca Ltd). Participants were rested in a supine position for 30 min during which their blood pressure was checked using a digital blood pressure monitor (A&D UA-767).

During RMR and DIT testing, participants were requested to minimise movements and remain awake throughout the measurement period. Participants could read or watch films on a laptop which, if required, was positioned on a small table sat over the bed. The Deltatrac and GEM measurements were collected with the participant in a supine position. ECAL measurements were collected with the participant in a semi-reclined position with a cushion supporting the mouthpiece connector, so it was not necessary for the participant to hold it. Participants were allowed to remove the mouthpiece momentarily during testing if they needed to swallow but were told to replace it as soon as possible. Temporary removal of the ECAL mouthpiece creates an abnormal airflow and consequently those data are not recorded. Room temperature was maintained throughout at between 22 and 24°C and blankets were available on request. All measurements were taken by the same trained investigator.

### Energy expenditure

RMR was determined by the Deltatrac, GEM and ECAL indirect calorimeters. The order of machines was randomly allocated on each test day using a random-order generator (Oxford Brookes University). V_CO2_ and V_O2_ data generated from the Deltatrac, GEM and ECAL were collected and RMR, fat oxidation, carbohydrate (CHO) oxidation and RQ were calculated using the Lusk equation^(^^26^^)^. The equations based on Lusk's calorific factors have been reported as more appropriate for use in normal conditions^(^^27^^)^. To allow for stabilisation with the mouthpiece or within the hood the first 10 min of every 30 min time period was discarded^(^^28^^)^. The averages of the remaining 20 min of data were used. A steady-state target CV for V_O2_ and V_CO2_ production during these 20 min was set as within 20 %^(^^24^^,^^29^^)^. This level was specified to account for the fluctuations observed in breath samples collected from a mouthpiece compared with samples collected from a ventilated hood.

Following the consumption of a 1553 kJ (371 kcal) meal, DIT was determined on the GEM and the Deltatrac in 15 min intervals over a 240 min period. DIT is related to meal size and 4 h has been reported as an adequate amount of time for 78 % of the DIT to be measured, increasing to 90 % in meals less than 3343 kJ^(^^30^^)^. The first 5 min of every 15 min collected was discarded to allow for stabilisation within the hoods^(^^28^^)^. The averages of the remaining 10 min of data were used for analysis. DIT was calculated as the increase in EE above RMR values for 4 h after meal intake. V_O2_ and V_CO2_ data generated from the Deltatrac and the GEM were collected and DIT measures were calculated using the Lusk equation^(^^26^^)^.

### Standard meal

The test meal consisted of 30 g cornflakes (Kellogg's) served with 125 ml semi-skimmed milk (Tesco British), 15 g strawberry milkshake powder (Nesquik, Nestlé) mixed with 200 ml semi-skimmed milk (Tesco British), 20 g medium-chain TAG (MCT) (Trec Nutrition) and 200 ml of tap water. The total energy content of the meal was 1553 kJ as a larger meal may have delayed the peak response and lengthened the total duration of DIT^(^^31^^)^. It has been shown that MCT consumption increases EE and fat oxidation^(^^32^^)^ and was added to ensure a significant rise in EE occurred postprandially. Twenty grams of MCT oil was mixed with the milkshake immediately before serving. This was based on previous research that found 20 g of MCT to have an increase in DIT of about 9·4 % of total test meal energy^(^^9^^)^.

### Statistical analyses

Outcome measures consisted of V_O2_, V_CO2_, EE (RMR and DIT), RQ, fat oxidation and CHO oxidation. Accuracy of the machines (inter-machine variability) was measured by comparison of RMR and DIT measures between machines using repeated measures ANOVA and paired samples *t*-test respectively. Repeatability of machines (intra-machine variability) was measured by comparing RMR and DIT measures collected from the same machines on test day 1 (T1) and test day 2 (T2) using paired-samples *t*-test. Significance was set at *P* < 0·05. The relative variability of measures was calculated by using the CV. Individual data were examined for the percentage difference between mean measures. The strength of relations was determined using Pearson's correlation coefficient and Bland Altman plots were used to determine agreement of machines compared with the Deltatrac. The 95 % limits of agreement were calculated as the mean difference plus or minus two standard deviations of the differences, within which 95 % of differences between measurements by the two methods are expected to lie.

For DIT measures, an average time point for each 30 min period on the same machine was calculated. For example, measures on the Deltatrac collected at 15–30 min were averaged to give one reading at 22·5 min and then subsequently at 52·5, 82·5, 112·5, 142·5, 172·5, 202·5 and 232·5 min. An *a priori* power calculation predicted that a sample size of nineteen volunteers would be sufficient to achieve 90 % power based on a sd of 264 kJ/d and a change in mean RMR of plus or minus 209 kJ/d^(^^15^^)^. The incremental area under the curve was determined for total DIT using the trapezoidal rule for values above the baseline RMR. The relative increment in EE at any time point compared with the baseline value was used to assess differences at each time.

Statistical analyses were performed using IBM SPSS Statistics 21.0 (2013; SPSS Inc.) and data and figures were processed in Microsoft Excel 2010. Values are presented as mean values plus or minus standard deviation or standard error of the mean.

## Results

The study was successfully completed by twenty participants on both test days. Analysis of steady-state data revealed that many of the ECAL sessions (*n* 10 V_O2_, *n* 11 V_CO2_) were not within the target 20 % CV. As this would have excluded a large amount of ECAL data and was inconsistent across all machines, it was decided that all results should be included. Therefore, RMR analysis includes data from all participants. DIT analysis includes data from nineteen participants as one participant's data were excluded due to values missing from the first 2 h of DIT measurement.

### RMR – inter-machine variability

Repeated measures ANOVA revealed statistically significant differences between machines for all measures (*P* < 0·05). *Post hoc* analysis with Bonferroni adjustment revealed that the GEM was statistically higher than the Deltatrac on T1 and T2 for V_CO2_, V_O2_, RMR and fat oxidation (*P* < 0·005) (Table 2) and statistically higher than the ECAL for V_CO2_ on T1 and T2 (*P* < 0·05) and RQ and CHO oxidation on T2 only. The ECAL was statistically higher than the Deltatrac for measures of V_O2_, RMR and CHO oxidation on T2 and, RQ and fat oxidation on both test days (*P* < 0·05). Variation was greatest on the ECAL with higher standard errors than the Deltatrac and the GEM on all measures.

**Table 2.** RMR inter-machine mean difference in all participants (*n* 20) for V_CO2_, V_O2_, RMR, RQ, CHO ox & FAT ox collected on the DT, ECAL and GEM, T1 and T2. Significance was calculated using repeated measures ANOVA with Bonferroni adjustment for multiple comparisons

	V_CO2_ (ml/min)						V_CO2_ (ml/min)						RMR (kJ/d)					
	DT-ECAL		GEM – DT		GEM – ECAL		DT-ECAL		GEM – DT		GEM – ECAL		DT-ECAL		GEM – DT		GEM – ECAL	
	Mean	se	Mean	se	Mean	se	Mean	se	Mean	se	Mean	se	Mean	se	Mean	se	Mean	se
T1	−1·54	9·74	33·00**	1·99	31·49*	9·97	−18·86	11·77	46·50**	2·25	27·62	12·04	−458·8	337·74	1314·00**	60·19	855·24	346·39
T2	−11·76	7·86	35·40**	2·90	23·67*	7·45	−41·86**	8·100	50·90**	3·72	9·07	7·79	−1064·47**	231·44	1434·20**	100·37	369·70	223·76

	RQ						CHO ox (kJ/min)						FAT ox (kJ/min)					

T1	0·06*	0·02	−0·03*	0·01	0·03	0·02	0·69	0·33	−0·01	0·13	0·67	0·32	−1·00*	0·36	0·92**	0·14	−0·08	0·35
T2	0·10*	0·02	−0·03	0·01	0·07*	0·02	1·04*	0·39	−0·03	0·18	1·00*	0·35	−1·78**	0·40	1·03**	0·20	−0·75	0·35

DT, Deltatrac; sem, standard error of mean; T1, test day 1; T2, test day 2; RQ, respiratory quotient; CHO ox, carbohydrate oxidation; FAT ox, fat oxidation.

Statistically different **P<0·005, *P < 0·05.

**Fig. 2.**
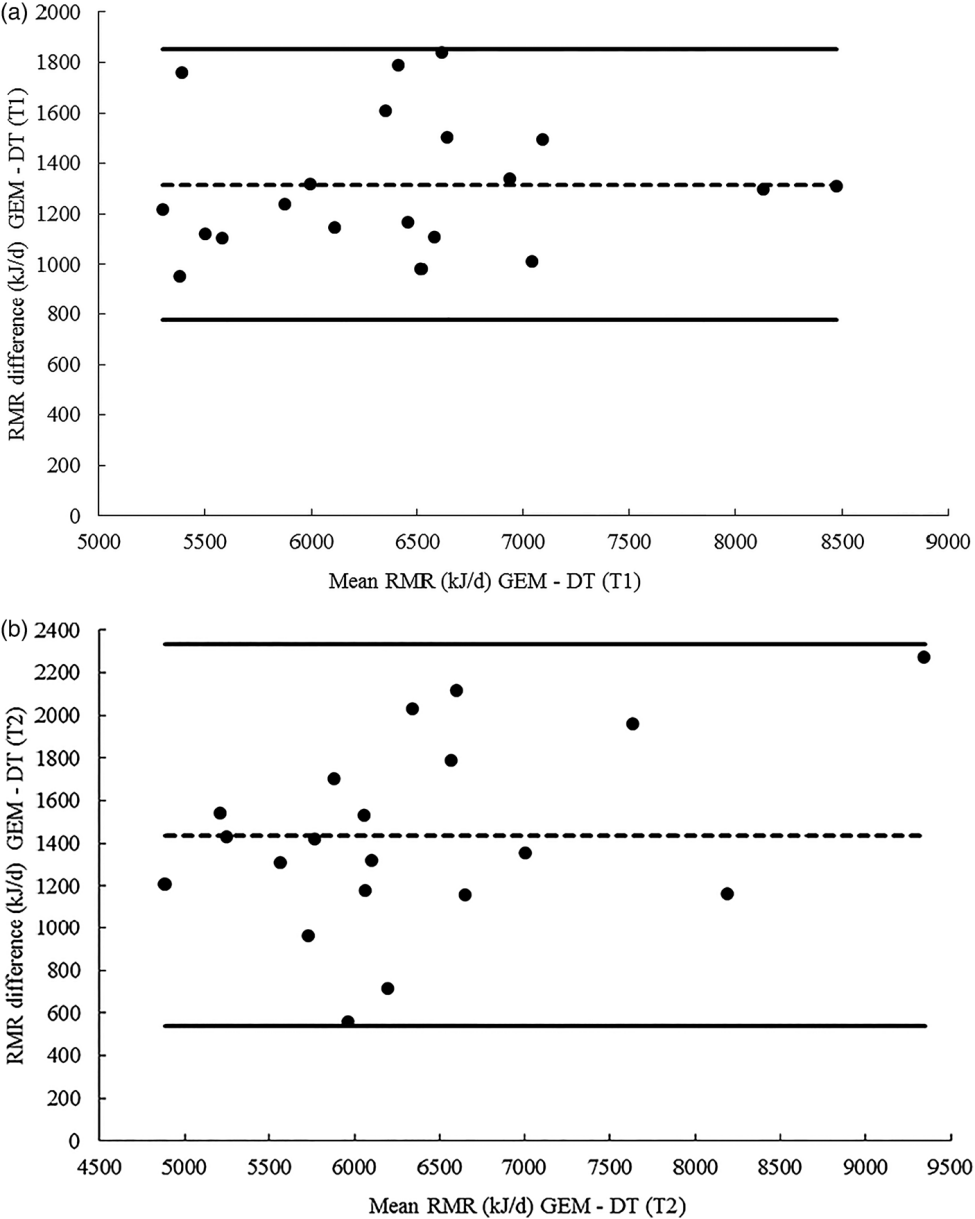
(a) Bland–Altman plot of mean difference in RMR measures collected on the GEM and the Deltatrac (DT) on test day 1 (T1). 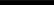 represents the upper and lower 95 % limits of agreement. 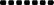 indicates the mean bias. The lower 95 % limit of agreement was 776 kJ/d and the upper 95 % limit of agreement was 1852 kJ/d. Difference was calculated as GEM minus DT. (b) Bland–Altman plot of mean difference in RMR measures collected on the GEM and the DT on test day 2 (T2). 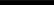 represents the upper and lower 95 % limits of agreement. 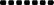 indicates the mean bias. The lower 95 % limit of agreement was 536 kJ/d and the upper 95 % limit of agreement was 2332 kJ/d. Difference was calculated as GEM minus DT.

**Fig. 3.**
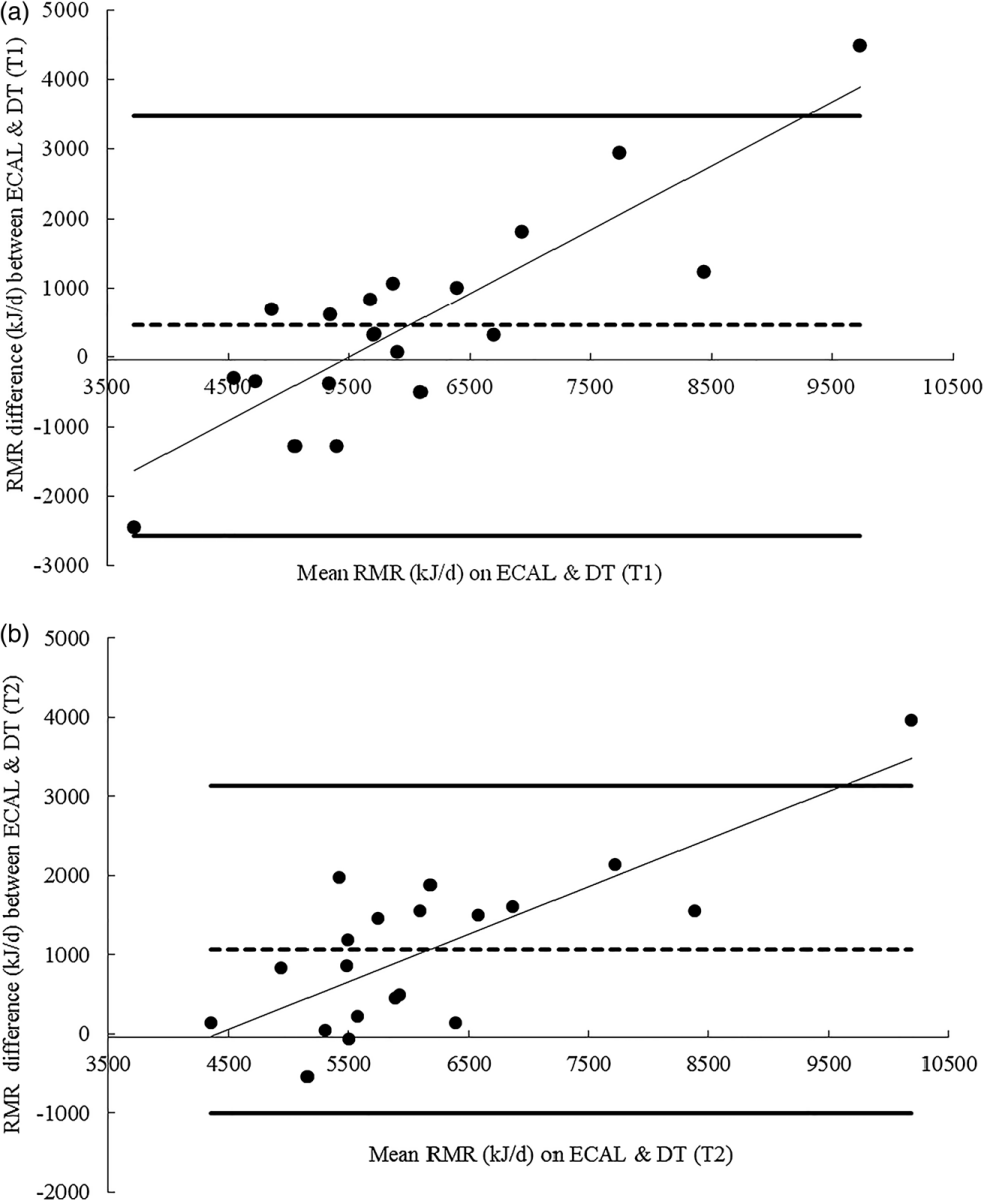
(a) Bland–Altman plot of mean difference in RMR measures collected on the ECAL and the Deltatrac (DT) on test day 1 (T1). 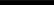 represents the upper and lower 95 % limits of agreement. 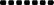 indicates the mean bias. The lower 95 % limit of agreement was −2562 kJ/d and the upper 95 % limit of agreement 3480 was kJ/d. Difference was calculated as ECAL minus DT. (b) Bland-Altman plot of mean difference in RMR measures collected on the ECAL and the DT on test day 1 (T2). 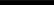 represents the upper and lower 95 % limits of agreement. 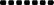 indicates the mean bias. The lower 95 % limit of agreement was −1006 kJ/d and the upper 95 % limit of agreement 3134 was kJ/d. Difference was calculated as ECAL minus DT.

Bland Altman analysis of RMR measures between the GEM and the Deltatrac indicated a consistent bias towards overestimation on the GEM with a mean difference of 1314 (sd 269) kJ/d on T1 and 1434 (sd 449) kJ/d on T2 (Fig. 2(a) and (b)). A smaller bias towards overestimation on the ECAL was observed with a mean difference of 459 (sd 1510) kJ/d on T1 and 1064 (sd 1035) kJ/d on T2. However, limits of agreement were wider and a proportional bias was observed suggesting that at higher RMR the difference between the two machines was greater (Fig. 3(a) and (b)).

### RMR – intra-machine variability

There were no significant differences between repeated measures of V_CO2_, V_O2_, RMR, RQ and substrate oxidation within the GEM or the Deltatrac. Within the ECAL only V_O2_ measures were significantly different between test days (*P* < 0·05). Significant correlations (*P* < 0·005) were observed for repeated measures of V_CO2_, V_O2_ and RMR on all machines (Table 3). Substrate oxidation and RQ were significantly correlated on the GEM (*P* < 0·05) and the ECAL (*P* < 0·005); however, this was in contrast to the Deltatrac for which low, non-significant correlations between RQ and substrate oxidation repeated measures were observed. Bland Altman analysis of RMR repeated measures estimated agreement within the GEM of 10 (sd 701) kJ/d (LOA −1391 and 1412 kJ/d) and within the Deltatrac of 130 (sd 489) kJ/d (LOA −848 and 1108 kJ/d). The greatest bias occurred within the ECAL with a mean difference of 475 (sd 1083) kJ/d and wide limits of agreement (LOA −2641 and 1691 kJ/d) (Table 4). The coefficient of variance was calculated as 4 (sd 5·3) % on the Deltatrac, 4·9 (sd 4·5) % on the GEM and 11·2 (sd 12·1) % on the ECAL. Differences within individuals repeated RMR measures were averaged to give a mean within individual difference of 5·4 % on the Deltatrac, 6·9 % on the GEM and 13·1 % on the ECAL.

**Table 3.** Correlations and significance of correlations for RMR measures on the DT, ECAL and GEM for V_CO__2_, V_O2_, RMR, RQ, CHO ox and FAT ox in all participants (*n* 20) on T1 and T2

	Intra-machine					
	DT		GEM		ECAL	
	T1, T2		T1, T2		T1, T2	
	*r*	*P*	*r*	*P*	*r*	*P*
V_CO2_ (ml/min)	0·83	0·005	0·81	0·005	0·8	0·005
V_O2_ (ml/min)	0·87	0·005	0·8	0·005	0·86	0·005
RMR (kJ/d)	0·87	0·005	0·8	0·005	0·85	0·005
RQ	−0·15	0·496	0·5	0·018	0·72	0·005
CHO ox (kJ/min)	0·06	0·766	0·56	0·009	0·71	0·005
FAT ox (kJ/min)	0·17	0·48	0·57	0·009	0·74	0·005

DT, Deltatrac; T1, test day 1; T2, test day 2; RQ, respiratory quotient; CHO ox, carbohydrate oxidation; FAT ox, fat oxidaton.

*r*, values of Pearson's correlation test. *P* values for statistical significance of correlation set at *P* < 0·05 and *P* < 0·005.

**Table 4.** Bland Altman analysis of RMR repeated measures in all participants (*n* 20) on T1 and T2. Agreement within machines (intra-machine variability) was calculated by comparing differences between repeated measures within the same machine (GEM, DT and ECAL)

	GEM				DT				ECAL			
	(T1, T2)				(T1, T2)				(T1, T2)			
	MD	sd	LOA		MD	sd	LOA		MD	sd	LOA	
V_CO2_ (ml/min)	−1·2	21·2	−43·5	41·1	1·2	16·9	−32·6	35·0	−9·0	36·6	−82·1	64·1
V_O2_ (ml/min)	0·8	24·3	−47·8	49·5	5·3	16·8	−28·2	38·8	−17·7	36·5	−90·7	55·3
RMR (kJ/d)	10·2	700·7	−1391·2	1411·6	130·3	489·1	−847·8	1108·4	−475·3	1083	−2641·3	1690·6
RQ	−0·01	0·05	−0·10	0·08	−0·01	0·05	−0·12	0·09	0·02	0·08	−0·14	0·19
CHO ox (kJ/min)	−0·14	0·73	−1·60	1·33	−0·16	0·78	−1·72	1·40	0·19	1·40	−2·61	3·00
FAT ox (kJ/min)	0·14	0·78	−1·41	1·7	0·25	0·73	−1·20	1·70	−0·52	1·30	−3·11	2·07

DT, Deltatrac; T1, test day 1; T2, test day 2; MD, mean difference; sd, standard deviation; LOA, limits of agreement; RQ, respiratory quotient; CHO ox, carbohydrate oxidation; FAT ox, fat oxidation.

### DIT – inter-machine agreement

**Fig. 4.**
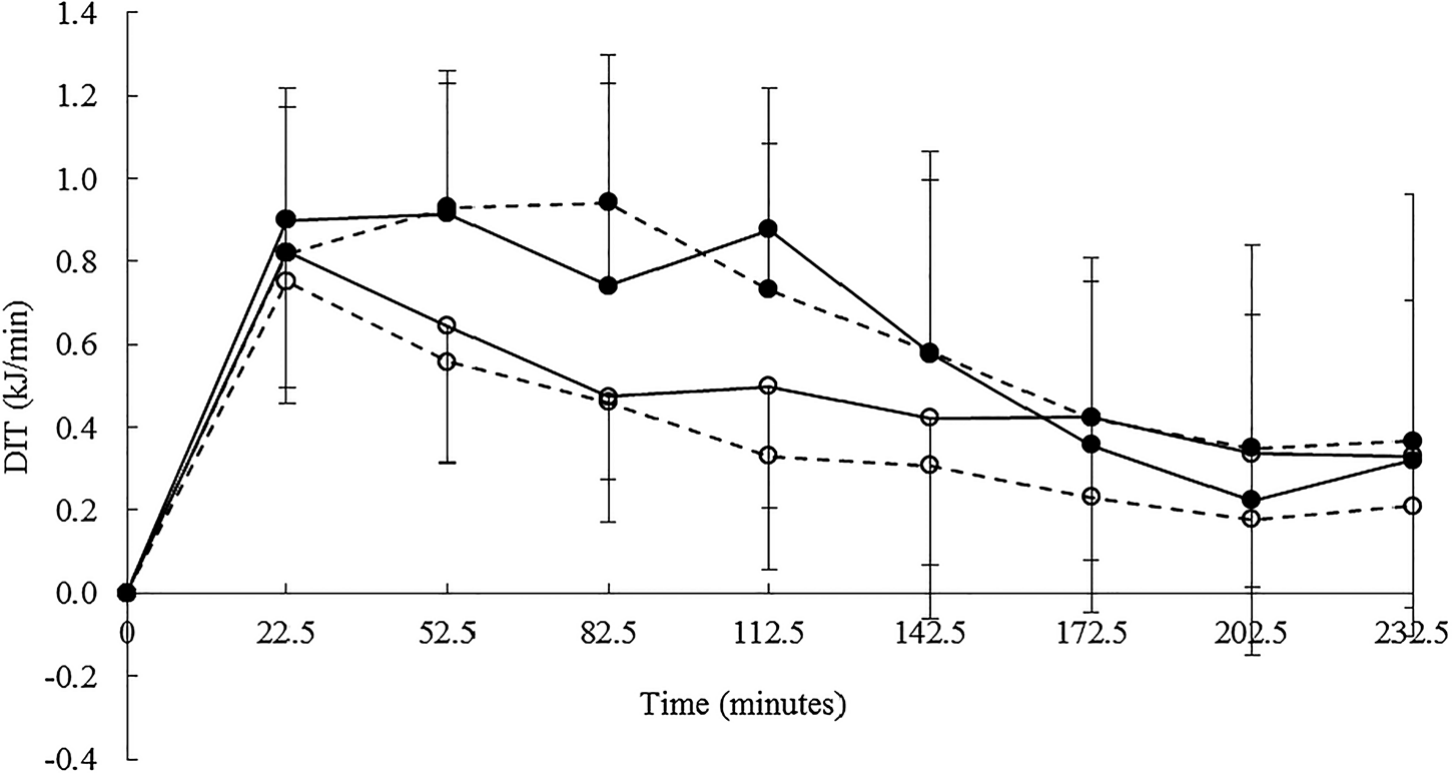
Mean diet-induced thermogenesis (DIT) response to a 1553 kJ meal over a 4 h period. Time zero indicates the pre-meal RMR measure. DIT was calculated as the total energy expenditure above RMR. An average time point for each 30 min period on the GEM and the Deltatrac (DT) was calculated as indicated on the *x* axis. The GEM is indicated by 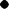 and the DT by 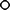. 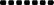 indicates test day 1 (T1) and 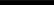 indicates test day 2 (T2). Values are means with standard deviations represented by vertical bars.

There was a significant correlation (*P* < 0·05) between the GEM and the Deltatrac for DIT measures over the 4 h period on T2 (*r* 0·49) but not on T1. Consistent with RMR measures, the GEM reported higher DIT values than the Deltatrac (Fig. 4); however, this was significant on T1 only (*P* < 0·005). DIT as a percentage of total EE was calculated as 9·1 (sd 3·2) % and 11·9 (sd 4·0) % on the Deltatrac and the GEM respectively (T1) and 11·4 (sd 5·0) % and 11·6 (sd 4·4) % on the Deltatrac and the GEM, respectively (T2). Bland Altman analysis of DIT measures indicated a small but consistent bias towards over estimation on the GEM of 63·3 (sd 63·6) kJ (LOA −63·9 and 190·4 kJ) on T1 and 33·6 (sd 56·5) kJ (LOA −79·4 and 146·5 kJ) on T2.

The same pattern of differences between the GEM and the Deltatrac were observed for V_O2_ and V_CO2_ with the GEM reporting consistently higher (60·5 (sd 6·7) ml/min (T1), 58·0 (sd 8·8) ml/min (T2), and 40·9 (sd 7·5) ml/min (T1), 38·5 (sd 7·8) ml/min (T2), respectively). Fat oxidation measures were significantly higher (*P* < 0·05) on the GEM (53·3 (sd 45·8) kJ) (T1), 34·6 (sd 49·5) kJ (T2); however, differences were NS between CHO oxidation measures 9·9 (sd 29·1) kJ (T1) and −1·1 (sd 26·3) kJ (T2).

### DIT – intra-machine variability

There were low, non-significant correlations between DIT repeated measures on the GEM and the Deltatrac despite no significant differences (*P* > 0·05). Bland Altman analysis estimated agreement within the Deltatrac as 22·3 (sd 63·2) kJ (LOA −148·7 and 104·1 kJ) and on the GEM of 7·4 (sd 79·8) kJ; however, limits of agreement on the GEM were wider (LOA −152·3 and 167·1 kJ). The coefficient of variance for intra-machine variability was calculated as 36 % on the Deltatrac and 30 % on the GEM.

There were no significant differences for repeated measures of fat oxidation or CHO oxidation (*P* > 0·05). There was a significant correlation (*P* < 0·05) on the GEM for CHO oxidation. Bland Altman analysis estimated less bias between repeated CHO measures on the GEM of 1·9 (sd 31·7) kJ (LOA −61·6 and 65·3 kJ) compared with the Deltatrac −9·2 (sd 35·8) (LOA −80·9 and 62·5 kJ) but a greater bias between repeated measures on the GEM for fat oxidation of 13·0 (sd 36·3) kJ (LOA −85·6 and 59·6 kJ) than the Deltatrac of 5·7 (sd 68·3) kJ (LOA −130·9 and 142·3 kJ).

## Discussion

The main finding of the present study was that the GEM and the ECAL were not accurate alternatives to the Deltatrac for measures of RMR or DIT. Measures were consistently higher on the GEM and higher and more variable on the ECAL. The reliability of the machines for repeated RMR measures was good, with the GEM and the Deltatrac CV within 5 %. The ECAL was higher at 11·2 %, however, if one participant's data were excluded this came down to within 10 %. DIT repeatability measures within the GEM and the Deltatrac were not significantly different; however, the CV was at least 30 % on both machines.

Owing to a lack of published data on the GEM and the novel aspect of the ECAL, it was not possible to make direct comparisons with previous validation research; however, comparable indirect calorimeters have been validated against the Deltatrac and it is these which are used here for comparison.

Three studies compared the QuarkRMR^®^ (Cosmed^®^), a hooded indirect calorimeter similar to the GEM, with the Deltatrac, reporting mixed conclusions^(^^13^^,^^14^^,^^22^^,^^29^^)^. All observed similar differences between mean RMR measures ranging from 100 to 163 (sd 460) kJ/d; however, the Quark was rejected as an accurate alternative by Graf *et al.*^(^^14^^)^ and Sundstrom *et al.*^(^^30^^)^ based on high limits of agreement (LOA −820 and 1021 and −1360 to 1799 kJ/d, respectively). In contrast, Blond *et al.*^(^^13^^)^ found the Quark's limits of agreement were within those observed on the Deltatrac and therefore accepted it as a valid alternative. Two of the aforementioned studies^(^^13^^,^^30^^)^ compared another hooded indirect calorimeter, the CCMExpress^®^ (Medgraphics^®^) to the Deltatrac and again found no agreement between methods. Similar to the GEM, the CCMExpress^®^ was found to overestimate compared with the Deltatrac. Sundstrom *et al.*^(^^30^^)^ reported unusually large differences on the CCMExpress^®^ of more than 4184 kJ/d, potentially due to measurement error. Graf *et al.*^(^^14^^)^ saw a smaller difference (464 (sd 544) kJ/d) than the present study but rejected it as an accurate alternative based on high limits of agreement (LOA −1536 and 628 kJ/d).

Of three validation studies^(^^12^^,^^14^^,^^16^^)^ comparing portable calorimeters which, similar to the ECAL, can measure V_O2_ and V_CO2,_ only one reported agreement with the Deltatrac^(^^12^^)^. The Cosmed K4 *b*^2^ facemask (Cosmed Srl) was rejected due to underestimating RMR by similar differences as were seen with the ECAL of 1121 (sd 2937) kJ/d^(^^16^^)^. The CCMExpress^®^ face mask underestimated by a small non-significant amount; however, as with the ECAL, there was high variability and it was rejected based on wide limits of agreement (21 (sd 841) kJ/d (LOA −1661 and 1703 kJ/d))^(^^14^^)^. The VO2000 facemask (Medgraphics) was the only machine accepted based on measures coming within 5 % of those on the Deltatrac^(^^12^^)^. The majority of comparison studies tend to use the MedGem RMR^®^, a portable calorimeter that uses a mouthpiece to measure VO_2_; however, they report mixed conclusions^(^^17^^)^. Frankenfield & Coleman^(^^17^^)^ reported that of eight portable calorimeter studies only two studies reported agreement to the Deltatrac, both reporting that this was based on there being low, non-significant differences between the machines^(^^18^^,^^19^^)^.

Of two validation studies comparing DIT measures from a hood and a mouthpiece to the Deltatrac, both concluded agreement between methods^(^^13^^,^^19^^)^. Blond *et al.*^(^^13^^)^ found agreement based on the magnitude of DIT (as a % of TEE) of 4·9 (sd 1·9) % on the Deltatrac and 4·9 (sd 2·0) % on the Quark^(^^13^^)^. Similarly, the present study saw a comparable magnitude of DIT values between the GEM (T1, T2) and the Deltatrac (T2) ranging from 11·4 (sd 5·0) to 11·9 (sd 4·0) %. This was higher than the theoretical magnitude of DIT, which is estimated at 7–9 % of EE^(^^28^^)^ despite the present study having a lower-energy content meal (1553 kJ *v.* 2872 kJ). The addition of MCT to the meal may explain some of this extra energy and has been shown to increase EE by about 9·4 % when used in these quantities^(^^9^^)^. St-Onge *et al.*^(^^19^^)^ used a 2510 kJ meal in their validation of the MedGem, which, despite a large over estimation by the MedGem of DIT measures (1448 kJ/d) they accepted as an accurate alternative to the Deltatrac based on a finding of no bias between measures of post-prandial EE (the mean of all EE measures after meal consumption).

Conflicting results between studies may be, in part, down to differences in study design. Some studies do not include a repeated measure^(^^14^^,^^18^^,^^19^^,^^21^^)^ or use healthy participants in a non-fasted state^(^^14^^)^. Comparisons made under non-standardised conditions make it even harder to determine if the variation is due to physiological factors or machine variation^(^^22^^)^. Additionally, some studies reject new methods without defining what an acceptable variation from the old method would be^(^^14^^,^^16^^,^^20^^,^^24^^,^^29^^)^. Setting limits for acceptable variation between indirect calorimeters is subjective, nevertheless, studies should at least provide a rationale for the basis of their conclusions.

Expectations for machines to agree are confounded by the variation that exists not only biologically within individuals, but also between the function and design of the machines. The adaptability of the mouthpiece allows it to be used in a wide range of settings, however, increased levels of discomfort, changes in breathing patterns and the energy cost of being in an upright position are suggested as some of the reasons for the increased values commonly observed in mouthpiece measures^(^^17^^–^^19^^)^. Using a facemask may have improved on some of these factors, however, as the ECAL was designed to be used with a mouthpiece the manufacturer's protocol was followed. Compher *et al.*^(^^28^^)^ summarised that the additional cost of a mouthpiece is about 293 kJ. This value is not consistent with the differences observed in the present study of 459 kJ (T1) and 1064 kJ (T2) suggesting the differences were due to additional factors. A mouthpiece can introduce the potential for leakages if the participant does not form a complete seal around it; however, the results gave no indication of this. Additionally, all participants successfully completed 30 min with relative ease.

When comparisons between indirect calorimeters and an established reference standard are made it is not possible to be certain which machine is reporting accurately, therefore, agreement of methods is measured with the assumption that the reference standard is accurate. Inaccessibility to new parts meant it was not possible to perform monthly methanol burns on the Deltatrac as a way to measure the accuracy of readings. Furthermore, the Deltatrac is an old machine and poor consistency between RQ values on repeated measures were observed, implying the possibility that the Deltatrac may not be a reliable reference, a conclusion also suggested in another, recent study^(^^33^^)^. It may be possible to compare trends between machines as the magnitude of difference should in theory be similar even if the values themselves are not (as observed on the GEM in the present study). Another, more consistent measure of a machine's validity is its reliability of repeated measures. Whether measuring individuals for research purposes or for health, it is the consistency of the machine that will enable changes over time to be measured. If the reliability of a machine cannot be proven this would suggest that agreement between methods is likely to be poor too^(^^34^^)^. Therefore, for every validation study both accuracy and repeatability should be measured (and regularly checked). However, studies that include both are limited. This is surprising as acceptable differences in repeated measures of RMR within the same individual are well established in the literature, with the Deltatrac variation reported to be within 5 %^(^^35^^,^^36^^)^. In a systematic review by Compher *et al.*^(^^28^^)^ they conclude that day-to-day subject variation ranges from 3–5 % over a 24 h period increasing to about 10 % when measured over weeks or months. When the present study calculated the mean difference on an individual level it found that overall mean differences in RMR on the GEM were 6·9 and 5·4 % on the Deltatrac. This was slightly above the 5 % observed in previous Deltatrac studies^(^^35^^–^^36^^)^ but within the 10 % variation observed in other studies of a similar length^(^^28^^)^. When the individual data was analysed further it was found that 50 % of measures on the GEM and 60 % of measures on the Deltatrac were within 5 %, increasing to 75 and 90 % respectively within 10 %.

A variation study comparing intra-individual variation of a portable calorimeter, the MOXUS Modular V_O2_ mouthpiece (AEL Technologies), to the Deltatrac was investigated by Roffey *et al.*^(^^15^^)^. They concluded that after 5 d of separate testing the CV of the MOXUS was 7·3 (sd 2·3) % *v.* 5·3 (sd 1·2) % on the Deltatrac. The present study found the CV of the ECAL to be 11·2 (sd 12·1) %, however, if one participant was excluded this came down to 8·9 (sd 6·5) %. When analyses of the individual differences on the ECAL were made it was found that 40 % of participants had repeated RMR measures within 10 %. Two other mouthpiece studies that looked at individual values reported 43 % of measures coming within 5 %^(^^17^^)^ and 80 % of measures coming within 10 %^(^^21^^)^.

Fewer studies measured DIT repeatability. A recent study by Ruddick-Collins *et al.*^(^^31^^)^ reported that the day-to-day variability of DIT measurements were high, with CV typically in the range of 15–29 % although in one study it was as high as 42 %. Despite the present study observing non-significant differences between DIT measures, CV on the Deltatrac was 36 and 30 % on the GEM. These values were similar to Ruddick-Collins *et al.*^(^^31^^)^ who reported a CV of 29 % in measurements lasting 4 h. It was unclear why CV values were so high. DIT calculations were based on RMR which, although produced much lower CV values, does introduce additional error into the calculation. Some studies have used a fixed RMR value in an attempt to overcome this issue with some seeing an improvement in measures and others not^(^^31^^)^. Despite using standardised methods, DIT is relatively small compared with RMR and therefore very sensitive to the noise associated with boredom, fidgeting and restlessness^(^^37^^,^^38^^)^. There may also have been an effect of familiarisation with the machines and procedures on the second day of testing which could explain some of the variation^(^^39^^)^.

Although the ECAL was designed for measuring RMR over a 10 min period, in the present study data were collected from subjects for 30 min to ensure consistency of protocol. To investigate the effect this may have had on the results the first 10 min were analysed separately (data not shown); however, there was no indication of any significant differences compared with the findings reported using the 20 min data. Towards the end of the study it emerged that RQ values on the ECAL had dropped below 0·70 on the final six sessions which is below physiological normal^(^^40^^)^. The ECAL was designed for health professionals assuming little technical expertise therefore the facility to perform a methanol burn are presently not included. Investigations by the researchers and manufacturers were unable to identify the cause of this therefore all subjects were included in the final analysis. Results excluding these participants were analysed separately (data not shown) and as expected, RQ values were no longer significantly different to the Deltatrac. There was also an improvement in repeatability measures with a CV of 8·1 %. The manufacturer is presently investigating the issue.

One limitation of the present study was the uneven number of male and female participants therefore the data between these two groups was not analysed separately. Some intra-individual variation can potentially be reduced by taking simultaneous measures; however, this was not attempted in the present study as there have been conflicting results using this method^(^^16^^)^. During DIT measures an attempt to correct for this was made by swapping the hood every 15 min, however, we cannot be sure whether this may have contributed to the restlessness of the participant although excluding the first 5 min of data should have controlled for this.

In conclusion, the present study found that the GEM and the ECAL were not accurate alternatives to the Deltatrac; however, it cannot be discounted that the Deltatrac itself may not have been a reliable comparison. This finding highlights the importance of improving the rigour of measures collected in comparison and validation studies^(^^33^^)^. The differences observed between machines support recommendations by previous studies that machines should not be used inter-changeably^(^^15^^,^^16^^)^. The present study recommends that all validation studies include a measure of repeatability as there is a greater consensus on intra-individual variation within the literature. Based on these measures the GEM can be advocated as a reliable alternative to the Deltatrac. The ECAL CV was within acceptable ranges (once data were excluded); however, technical issues with the machine demonstrated by the decreased RQ values suggest further research may be needed to ensure its repeatability can be maintained.
